# Preoperative systemic inflammation predicts postoperative infectious complications in patients undergoing curative resection for colorectal cancer

**DOI:** 10.1038/sj.bjc.6604997

**Published:** 2009-03-24

**Authors:** L H Moyes, E F Leitch, R F McKee, J H Anderson, P G Horgan, D C McMillan

**Affiliations:** 1 University Department of Surgery, Faculty of Medicine-University of Glasgow, Royal Infirmary, Glasgow G31 2ER, UK

**Keywords:** colorectal cancer, surgery, glasgow prognostic score, infectious complications

## Abstract

The presence of systemic inflammation before surgery, as evidenced by the glasgow prognostic score (mGPS), predicts poor long-term survival in colorectal cancer. The aim was to examine the relationship between the preoperative mGPS and the development of postoperative complications in patients undergoing potentially curative resection for colorectal cancer. Patients (*n*=455) who underwent potentially curative resections between 2003 and 2007 were assessed consecutively, and details were recorded in a database. The majority of patients presented for elective surgery (85%) were over the age of 65 years (70%), were male (58%), were deprived (53%), and had TNM stage I/II disease (61%), had preoperative haemoglobin (56%), white cell count (87%) and mGPS 0 (58%) in the normal range. After surgery, 86 (19%) patients developed a postoperative complication; 70 (81%) of which were infectious complications. On multivariate analysis, peritoneal soiling (*P*<0.01), elevated preoperative white cell count (*P*<0.05) and mGPS (*P*<0.01) were independently associated with increased risk of developing a postoperative infection. In elective patients, only the mGPS (OR=1.75, 95% CI=1.17–2.63, *P*=0.007) was significantly associated with increased risk of developing a postoperative infection. Preoperative elevated mGPS predicts increased postoperative infectious complications in patients undergoing potentially curative resection for colorectal cancer.

Colorectal cancer remains the second most common cause of cancer death in Western Europe (Cancer Research UK, 2004). Despite advances in surgical techniques, perioperative care and adjuvant chemoradiotherapy, overall survival remains poor with only 50% patients surviving 5 years after potentially curative resection (McArdle and Hole, 2002).

Although tumour stage has been the main basis for predicting long-term survival in patients undergoing surgery for cancer, it is now recognised that postoperative complications contribute to poor cancer-specific survival (Rizk *et al*, 2004; Khuri *et al*, 2005; McArdle *et al*, 2005; Law *et al*, 2007). In particular, McArdle *et al* (2005) reported that in a prospective cohort of 2235 patients undergoing potentially curative resection for colorectal cancer, the postoperative complication of anastomotic leakage was associated with poorer cancer-specific survival, independent of Dukes stage. Law *et al* (2007) reported similar results in 1657 patients who underwent curative resection for colorectal cancer.

The basis of this observation is not clear. It has been postulated that the presence of an enhanced systemic inflammatory response may be responsible (McArdle *et al*, 2005), as the systemic inflammatory response, as evidenced by an elevated C-reactive protein concentration, has been shown to be associated with poor cancer-specific survival, independent of tumour stage (Nielsen *et al*, 2000; McMillan *et al*, 2003; Crozier *et al*, 2006). Studies have shown that elevated C-reactive protein concentrations in the postoperative period may predict an increased chance of postoperative infection (Welsch *et al*, 2007) and anastomotic leakage (Matthiessen *et al*, 2008). An initial appraisal might suggest that early postoperative infection initially declares itself as a rise in C-reactive protein before overt clinical infection develops, and this infection causes decreased long-term survival. However, it has been reported that the preoperative, but not the immediate postoperative, elevated C-reactive protein concentrations are associated with cancer-specific survival (Crozier *et al*, 2007).

With respect to the measurement of the systemic inflammatory response the combination of C-reactive protein and albumin (glasgow prognostic score (mGPS)) has been shown to improve the prediction of cancer-specific survival in a variety of common solid tumours (McMillan, 2008) including primary operable colorectal cancer (McMillan *et al*, 2007; Ishizuka *et al*, 2007). To our knowledge, the relationship between the preoperative systemic inflammatory response and postoperative complications has not been examined previously.

The hypothesis underlying this study was that a preoperative systemic inflammatory response, as evidenced by the mGPS, may be an underlying host characteristic that predisposes to postoperative infection in patients undergoing potentially curative resection for colorectal cancer.

## Patients and Methods

### Patients

Patients with histologically proven colorectal cancer who, on the basis of laparotomy findings and/or preoperative abdominal computed tomography, were considered to have undergone a potentially curative resection and had routine laboratory measurement of white cell counts, albumin and C-reactive protein, between January 2003 and October 2007, at Glasgow Royal Infirmary, were prospectively included in the study. The antibiotic regimen was based on 1 g second-generation cephalosporin and 500 mg metronidazole at induction of general anaesthesia. No other antibiotics were normally given. Three consultant surgeons (Anderson, Horgan and McKee) performed the majority of the operations (79%). The surgical approach was open in all cases.

Patients were assessed consecutively and their details were recorded in our colorectal cancer database. The tumours were staged according to the TNM criteria (American Joint Committee on Cancer Colon and rectum, 2002). Patients who had preoperative clinical evidence of infection or other inflammatory conditions were excluded from the study. A total of 23 patients were excluded on the basis of these criteria.

The extent of deprivation was defined using the Carstairs deprivation index (Carstairs and Morris, 1991). This is an area-based measure derived from the 2001 census, using the postcode of residence at diagnosis, which divides the score into a seven-point index. For illustrative purposes, these results are presented by amalgamating the seven categories into three groups: affluent (categories 1 and 2), intermediate (categories 3–5) and deprived (categories 6 and 7). The Carstairs deprivation index has been extensively utilised in cancer patients, and is particularly appropriate for use in the central belt of Scotland (Hole and McArdle, 2002).

Patients were assessed for the following complications: infectious (wound infection, intra-abdominal abscess, anastomotic leak, pneumonia and septicaemia) and noninfectious (cardiac events encompassing acute coronary syndrome and acute myocardial infarction and pulmonary embolism). The criteria used to define infectious complications were the same as that described previously (Ytting *et al*, 2005). (1) Wound infection was defined as the presence of pus, either discharged spontaneously or requiring drainage. Wound infection included a subgroup of patients with perineal infection, following abdominoperineal resection of the rectum. (2) Intra-abdominal abscess was verified by either surgical drainage or by ultrasonographically guided aspiration of pus. (3) Anastomotic leakage was defined as radiologically verified fistula to bowel anastomosis or diagnosed by relaparatomy. (4) Pneumonia was defined by fever above 38.5°C and a positive X-ray, and requirement of antibiotic treatment. (5) Septicaemia was defined by clinical symptoms combined with a positive blood culture.

Non-symptomatic or minor urinary tract infection was not recorded, and therefore only included if complicated by septicaemia.

The study was approved by the Research Ethics Committee, Royal Infirmary, Glasgow.

### Blood parameters

Routine preoperative laboratory measurements of white cell count, albumin and C-reactive protein concentration were carried out. The coefficient of variation for these methods, over the range of measurement, was <10% as established by routine quality control procedures.

The mGPS was constructed as described previously (McMillan *et al*, 2007). Briefly, a normal albumin (>35 g l^−1^) and normal C-reactive protein (<10 mg l^−1^) scores 0, with an elevated C-reactive protein scoring 1 and an elevated C-reactive protein together with a low albumin scoring 2.

### Statistics

Data are presented as median (range). Grouping of the variables was carried out using standard thresholds. Comparisons between groups of patients were carried out using the Mantel–Haenszel (*χ*^2^) test for trend as appropriate. Logistic regression analysis was used to examine the effect of variables on the development of postoperative infection. Analysis was performed using SPSS software (SPSS for Windows Version 15.0, SPSS Inc., Chicago, IL, USA).

## Results

The relationship between the preoperative systemic inflammatory response (mGPS) and clinicopathological characteristics in patients (*n*=455) who underwent curative surgery for colorectal cancer is shown in Table 1. The majority of patients were over the age of 65 years (70%), were male (58%), were deprived (53%), and presented for elective surgery (85%). The majority of patients presented with colonic tumours (60%) had T3–4 disease (81%), no nodal involvement (61%) and had TNM stage I/II disease (61%). The majority of patients had preoperative haemoglobin (56%), white cell count (87%) and mGPS 0 (58%) in the normal range.

After surgery, 86 (19%) patients developed a postoperative complication; 70 (81%) of which were infectious complications. The 16 non-infectious events were pulmonary embolism (*n*=1), myocardial infarction (*n*=6), acute coronary syndrome (*n*=5) and acute onset atrial fibrillation (*n*=4). The 70 infectious events were septicaemia (*n*=6), pneumonia (*n*=33), anastomotic leak (*n*=14), intra-abdominal collection (*n*=2) and wound infection (*n*=15).

An elevated mGPS was associated with deprivation (*P*<0.05), emergency presentation (*P*<0.001), peritoneal soiling (*P*⩽0.01) colonic tumours (*P*<0.001), advanced tumour stage (*P*<0.01), low haemoglobin (*P*<0.001), high white cell count (*P*<0.001) and postoperative infections (*P*<0.01).

The relationship between clinicopathological characteristics, systemic inflammatory response and postoperative infections in patients undergoing surgery for colorectal cancer is shown in Table 2. On binary logistic regression analysis, deprivation (*P*<0.10), emergency presentation (*P*<0.001), peritoneal soiling (*P*=0.001), elevated preoperative white cell count (*P*<0.001), C-reactive protein (*P*<0.001), albumin (*P*<0.001) and mGPS (<0.001) were associated with increased risk of developing a postoperative infection. On multivariate analysis, peritoneal soiling (*P*<0.01), elevated preoperative white cell count (*P*<0.05) and mGPS (*P*<0.01) were independently associated with increased risk of developing a postoperative infection.

When only those patients who presented electively were considered in the analysis (*n*=385), on binary logistic regression analysis, only the mGPS (OR=1.75, 95% CI=1.17–2.63, *P*=0.007) was significantly associated with increased risk of developing a postoperative infection.

## Discussion

The results of this study show that the preoperative systemic inflammatory response, as evidenced by the mGPS, was independently associated with an increased risk of postoperative infectious complications in patients undergoing potentially curative resection for colorectal cancer. This relationship persisted after patients with an emergency presentation were excluded from the logistic regression analysis. Given that postoperative infections are relatively common in patients undergoing surgery for colorectal cancer and are associated with increased hospital stay and treatment costs, it may be that the simple routinely available preoperative measurement of the mGPS will be clinically useful in identifying patients at high risk of developing infectious complications.

The basis of the independent relationship between an elevated mGPS before surgery and postoperative infections in patients with primary operable colorectal cancer is not clear. A plausible explanation is that an elevated mGPS may reflect compromised cell-mediated immunity as C-reactive protein is associated with lymphopenia (Nozoe *et al*, 2000; Leitch *et al*, 2007) and an impaired T-lymphocytic response (Canna *et al*, 2005) in patients with colorectal tumours. Alternatively, C-reactive protein is also associated with components of the innate immune system, including complement, and this response may also be compromised (Du Clos and Mold, 2004; Ytting *et al*, 2006). Also, an mGPS of 2, based as it is on the presence of an ongoing systemic inflammatory response and hypoalbuminaemia, reflects the loss of lean tissue and protein (McMillan *et al*, 1998; McMillan *et al*, 2001), which is likely to further compromise immune function. Therefore, the results of this study suggest that compromised immune function may occur before surgery and influences postoperative infectious complications.

It was of interest that, in this study, an elevated preoperative white cell count was also independently associated with increased risk of postoperative infectious complications. However, the white cell count was strongly associated with mGPS, and compared with the mGPS, the significance of its association weakened on multivariate analysis. Furthermore, in those patients who presented electively, white cell count was no longer significantly associated with postoperative infection. This would support the routine use of the mGPS to identify patients at increased risk of developing infectious complications. However, it remains to be determined whether the preoperative systemic inflammatory response may be moderated and whether such moderation may reduce postoperative infectious complications.

In summary, the results of this study indicate that a simple inflammation-based prognostic score identifies patients at increased risk of developing infectious complications following potentially curative resection for colorectal cancer.

## Figures and Tables

**Table 1:** The relationship between the preoperative systemic inflammatory response (mGPS) and clinicopathological characteristics in patients undergoing potentially curative surgery for colorectal cancer (*n*=455)

	**mGPS 0**	**mGPS 1**	**mGPS 2**	
	***n*=259 (%)**	***n*=130 (%)**	***n*=66 (%)**	***P*-value**
*Age*				
<65 years	88 (34)	28 (28)	22 (33)	
65–74 years	88 (34)	46 (35)	18 (27)	
⩾75 years	83 (32)	48 (37)	26 (40)	0.283
*Sex*				
Female	98 (38)	62 (48)	30 (46)	
Male	161 (62)	68 (52)	36 (54)	0.102
*Deprivation*				
Affluent (1, 2)	15 (6)	1 (1)	2 (3)	
Intermediate (3, 4, 5)	118 (46)	53 (40)	24 (36)	
Deprived (6, 7)	126 (48)	76 (59)	40 (61)	0.014
*Presentation*				
Elective	245 (95)	100 (77)	40 (61)	
Emergency	14 (5)	30 (23)	26 (39)	<0.001
*Peritoneal soiling*				
None or serous fluid	256 (98%)	126 (97%)	61 (92%)	
Local pus	2 (1%)	4 (3%)	4 (6%)	
Free pus or faeces	1 (1%)	0 (0%)	1 (2%)	0.010
*Tumour site*				
Colon	128 (49)	93 (72)	54 (82)	
Rectum	131 (51)	37 (28)	12 (18)	<0.001
*Tumour*				
T1	24 (9)	3 (2)	0 (0)	
T2	48 (19)	9 (7)	4 (6)	
T3	128 (49)	69 (53)	33 (50)	
T4	59 (23)	49 (38)	29 (44)	<0.001
*Nodal involvement*				
N0	164 (63)	75 (58)	37 (56)	
N1	72 (28)	34 (26)	22 (33)	
N2	23 (9)	21 (16)	7 (11)	0.148
*TNM stage*				
I	63 (24)	10 (8)	4 (6)	
II	102 (39)	68 (52)	33 (50)	
III	94 (37)	52 (40)	29 (44)	0.001
*Haemoglobin*				
⩾ 12g per 100 ml	180 (70)	64 (49)	11 (17)	
<12 g per 100 ml	79 (30)	66 (51)	55 (83)	<0.001
*White cell count*				
<8.5 × 10^9^/l	191 (74)	63 (49)	26 (39)	
8.5–11.0 × 10^9^/l	52 (20)	42 (32)	24 (37)	
>11 × 10^9^/l	16 (6)	25 (19)	16 (24)	<0.001
*Complications*				
None	223 (86)	105 (81)	41 (62)	
Infectious	25 (10)	21 (16)	24 (36)	
Non-infectious	11 (4)	4 (3)	1 (2)	0.004

**Table 2:** The relationship between the studied variables and postoperative infection in patients undergoing potentially curative surgery for colorectal cancer (*n*=455).

	**Univariate odds ratio (95% CI)**	***P*-value**	**Multivariate odds ratio (95% CI)**	***P*-value**
Age (<65/65–74/⩾75 years)	1.01 (0.74–1.38)	0.961		
Sex (female/male)	1.26 (0.74–2.12)	0.395		
Deprivation (1–2/3–5/6–7)	1.51 (0.94–2.42)	0.089		0.335
Presentation (elective/emergency)	3.52 (1.96–6.32)	<0.001		0.058
Peritoneal soiling (none or serous fluid/local pus/free pus or faeces)	7.17 (2.35–21.86)	0.001	4.91 (1.60–15.08)	0.005
Tumour site (colon/rectum)	1.02 (0.61–1.72)	0.935		
Tumour (T1/T2/T3/T4)	1.19 (0.86–1.64)	0.295		
Nodal involvement (N0/N1/N2)	0.99 (0.68–1.43)	0.942		
TNM stage (I/II/III)	0.97 (0.68–1.38)	0.844		
Haemoglobin (⩾12/<12 g per 100 mll)	1.53 (0.92–2.55)	0.104		
White cell count (<8.0/8.5–11.0/>11 × 10^9^/l)	1.95 (1.41–2.72)	<0.001	1.46 (1.01–2.11)	0.043
C-reactive protein (⩽10/>10 mg l^−1^)	2.79 (1.64–4.74)	<0.001		
Albumin (⩾35/<35 g l^−1^)	3.14 (1.80–5.48)	<0.001		
mGPS (0/1/2)	2.28 (1.64–3.16)	<0.001	1.76 (1.22–2.55)	0.003
